# Nanotubular networks in the aging brain: from neuroprotection to neurodegeneration

**DOI:** 10.3389/fnhum.2026.1864090

**Published:** 2026-06-26

**Authors:** Maya S. Jategaonkar, Nathan H. Miller, Skyler E. Zur, Changiz Geula, Peter Penzes, Colleen R. Zaccard

**Affiliations:** 1 Department of Neuroscience, Feinberg School of Medicine, Northwestern University, Chicago, IL, United States; 2 Mesulam Institute for Cognitive Neurology and Alzheimer's Disease, Feinberg School of Medicine, Northwestern University, Chicago, IL, United States; 3 Department of Psychiatry and Behavioral Sciences, Feinberg School of Medicine, Northwestern University, Chicago, IL, United States

**Keywords:** aging, amyloid-β, dendritic nanotubes, intercellular communication, microglia, neurodegeneration, tunneling nanotubes, α-synuclein

## Abstract

Intercellular communication in the central nervous system extends far beyond classical synaptic transmission. Contemporary studies utilizing *in vivo*, super-resolution, and ultrastructural microscopy approaches have revealed dynamic nanotubular networks capable of direct protein, organelle, and Ca^2+^ signal exchange between cells. Microglial tunneling nanotubes (TNTs) support cooperative α-synuclein aggregate clearance and mitochondrial rescue, whereas dendritic nanotubes (DNTs) mediate neuron-to-neuron connectivity that may contribute to amyloid-β redistribution or accumulation before plaque formation. Emerging evidence further suggests that nanotubular functions are dependent on cell type and structure. Microglial TNTs have typically been associated with aggregate handling, clearance, and cellular rescue, whereas neuronal TNTs and recently described DNTs provide distinct examples of disease-relevant cargo redistribution. A central unresolved question is not simply whether nanotubular networks mediate clearance and rescue or facilitate pathological spread, but how their regulation changes during development, adulthood, aging, and neurodegenerative disease. Existing studies emphasize disruption or collapse of TNT and DNT networks as important features of disease progression. Here, we propose that aging may contribute to intermediate states of nanotubular dysfunction before overt collapse, altering the efficiency, directionality, or consequences of intercellular exchange in a cell-type-specific manner. Mapping the cellular and regional dynamics of nanotubular networks in the brain, particularly during aging and Alzheimer’s disease, will be essential for determining when nanotubular connectivity supports resilience, becomes dysfunctional, or contributes to pathological protein redistribution. These efforts may reveal therapeutic strategies for preserving nanotubular integrity and function before the development of late-stage neurodegenerative disease.

## Introduction

Traditional models of cognitive decline have long centered on the progressive loss of synaptic integrity and chemical neurotransmission. Recent discoveries now broaden this view by identifying nanotubular connections as direct routes of communication among neural and glial cells. Emerging evidence suggests that the central nervous system (CNS) also relies on a complementary layer of intercellular connectivity from tunneling nanotubes (TNTs) (Budinger and Heneka, 2025). Reviews of these structures in astrocyte-neuron and glial cell communication further suggest that TNTs can mediate physiological signaling, organelle exchange, cellular stress responses, and neurodegenerative disease-relevant cargo transfer (Dias et al., 2026; Xu W. et al., 2026). Together, these observations place nanotubular connectivity at the intersection of intercellular stress handling, metabolic support, and pathological cargo transfer in neurodegenerative disease.

TNTs were first described in 2004 as long, ultra-thin membranous bridges connecting distant PC12 cells, a rat pheochromocytoma-derived neuronal cell line (Rustom et al., 2004). That same year, TNT-like structures were observed forming between immune cells, including natural killer cells, macrophages, and B cells (Onfelt et al., 2004). Subsequent work demonstrated that TNTs mediate rapid calcium (Ca^2+^) signaling between dendritic cells and monocytes, establishing TNTs as functional conduits in myeloid lineage cells (Watkins and Salter, 2005). Further investigations revealed structural heterogeneity, including thin F-actin-containing TNTs that support transfer of diverse cargo and signals, such as vesicles, proteins, pathogens, and Ca^2+^, and thicker TNTs containing both F-actin and microtubules that can support larger organelle transfer, including mitochondria (Onfelt et al., 2006; Abounit and Zurzolo, 2012; Zaccard et al., 2016). TNTs also differ in whether they establish open-ended cytoplasmic continuity or junctional contacts at the target-cell interface, which can support receptor-ligand interactions, electrical coupling, or second messenger-mediated Ca^2+^ signaling (Wang et al., 2010; Lock et al., 2016).

The functional relevance of nanotubular networks within the complex CNS environment has only recently come into focus, in part because their transient nanoscale architecture has hindered detection within the dense neuropil. Current studies indicate that microglial TNTs support rescue by accepting pathological protein burden for degradation while donating healthy mitochondria to stressed cells. This paired mechanism was first shown for α-synuclein redistribution among microglia and later extended to α-synuclein- and tau-burdened neurons, with complementary evidence for TNT-like neuron–microglia connections in brain tissue (Scheiblich et al., 2021; Scheiblich et al., 2024). Supporting *in vitro* evidence further shows that TNTs between neuron- and microglia-like cell lines can mediate bidirectional transfer of α-synuclein and mitochondria (Chakraborty et al., 2023). In neuronal and neuron-like cultures, TNTs have been shown to propagate neurodegenerative disease-associated proteins, including prions, tau, and amyloid-β (Gousset et al., 2009; Tardivel et al., 2016; Zhang et al., 2021). Together, these findings suggest that nanotubular networks may support either resilience or vulnerability in the brain, depending on their cellular origin, structure, and cargo.

Published commentary has highlighted aggregate-driven disruption of nanotubular networks as one potential contributor to neurodegenerative progression (Budinger and Heneka, 2025). Aging is accompanied by changes in cytoskeletal integrity and membrane organization, chronic low-grade inflammation, and cellular dysfunction (Lopez-Otin et al., 2013; Franceschi et al., 2018; Lai and Wong, 2020). Because TNTs and DNTs depend on cytoskeletal remodeling, membrane dynamics, and cellular stress responses, these age-associated processes may shape nanotubular connectivity in a context-dependent manner. Determining how aging influences these networks will be essential for understanding when nanotubular connectivity supports intercellular rescue, becomes structurally compromised, or contributes to maladaptive cargo redistribution. This Perspective synthesizes evidence from *in vitro*, *ex vivo*, and *in vivo* models to evaluate the emerging roles of TNTs and DNTs in neurodegenerative disease and aging.

## Microglial tunneling nanotubes in aggregate clearance and cellular rescue

**Figure 1:**
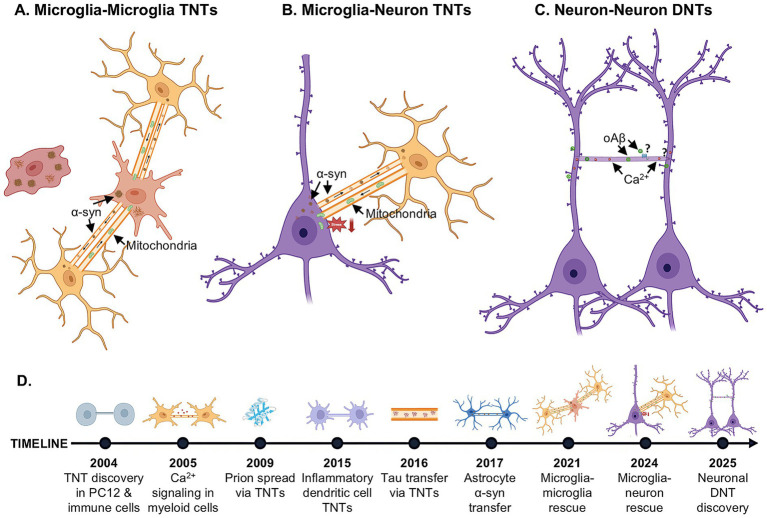
Recently identified nanotubular networks in microglia and neurons. **(A)** Microglia–microglia TNTs mediate cooperative burden sharing, including redistribution of pathological cargo, such as alpha-synuclein (α-syn), from overburdened microglia to healthy lower-burden cells, as well as mitochondrial donation from healthy to overburdened microglia. **(B)** A microglia–neuron TNT connects a microglial cell with a neuronal soma, supporting bidirectional rescue through aggregate transfer from the neuron to microglia and mitochondrial transfer from healthy microglia back to the neuronal soma. **(C)** Neuronal DNTs are short neuron-to-neuron connections between adjacent dendrites that may support Ca^2+^ signaling and pathological cargo transfer, including oligomeric amyloid-β (oAβ). Question marks highlight proposed but unresolved mechanisms of DNT-mediated transfer, including possible oAβ surfing along the DNT surface and junctional mechanisms for oAβ transfer or Ca^2+^ signal propagation. **(D)** Timeline of major nanotubular discoveries in immune and neuronal cell systems from 2004 to early 2026. Illustrations are schematic and not to scale. Figure was created using Biorender.com.

At the neuroimmune interface, microglial TNTs may provide on-demand intercellular networks that coordinate responses to proteotoxic stress. One important aspect of microglial TNT function is the cooperative handling of aggregated α-synuclein, a hallmark pathology of synucleinopathies such as Parkinson’s disease and a major focus of TNT-mediated transfer studies (Srinivasan et al., 2021). Under physiological conditions, microglia can phagocytose extracellular α-synuclein and target it for lysosomal degradation, including selective autophagy (Choi et al., 2020). When aggregate accumulation exceeds the degradative capacity of individual cells, TNTs enable overburdened microglia to redistribute intracellular α-synuclein to adjacent, lower-burden microglia for degradation while receiving mitochondrial support from healthy microglia (Figure 1A) (Scheiblich et al., 2021). This paired exchange expands the aggregate-processing capacity of local microglial populations and may reduce lysosomal overload. A similar rescue mechanism has been described in microglia–neuron interactions. Under proteotoxic or metabolic stress, microglial TNTs can connect with neuronal somata, enabling transport of aggregated α-synuclein or tau from neurons to microglia while supporting mitochondrial delivery back to stressed neurons (Figure 1B) (Scheiblich et al., 2024). This metabolic support may be especially important for neuronal activity, where efficient oxidative phosphorylation is required to sustain electrophysiological function (Harris et al., 2012).

Microglial TNT-based rescue is vulnerable to genetic risk variants relevant to neurodegeneration. The Parkinson’s disease-associated LRRK2 G2019S mutation impairs TNT formation, reduces microglia–microglia aggregate transfer, and leaves mutant cells with reduced degradative efficiency and persistent intracellular α-synuclein accumulation (Scheiblich et al., 2021). In microglia–neuron TNTs, LRRK2 G2019S and TREM2 variants linked to Alzheimer’s disease similarly impair TNT-mediated aggregate extraction and mitochondrial delivery to neuronal somata, reducing microglial support of neuronal bioenergetics (Scheiblich et al., 2024). These findings suggest that genetic disruption of microglial TNT networks may compromise both intercellular burden sharing and neuronal rescue, thereby increasing vulnerability to neurodegenerative disease.

Astrocyte and astrocyte–microglia co-culture studies extend this aggregate-handling model beyond microglia, while underscoring that intercellular transfer is not necessarily equivalent to clearance. In cultured human astrocytes, aggregated α-synuclein induces TNT formation and transfer to neighboring astrocytes, suggesting a route for pathological cargo redistribution, rather than demonstrating efficient degradation or rescue (Rostami et al., 2017). In human iPSC-derived astrocyte–microglia co-cultures, α-synuclein and amyloid-β aggregate degradation is increased relative to monocultures, and live imaging shows direct astrocyte–microglia contacts through TNTs and other membrane structures, supporting a model wherein microglia serve as degradative partners for aggregate-burdened astrocytes (Rostami et al., 2021). More recently, α-synuclein protofibrils were shown to induce transient TNT biogenesis in astrocytes, with TNT-associated intercellular transfer linked to reduced organelle stress, reactive oxygen species, and senescence markers (Raghavan et al., 2024). Together, these studies suggest that astrocyte-related TNT communication may contribute to either pathological redistribution or stress-associated aggregate handling, with the net outcome likely depending on cargo burden, cellular partners, and the degradative or metabolic capacity of recipient cells.

These studies point to a central unresolved issue: whether TNT-mediated transfer promotes clearance and rescue or instead redistributes pathological cargo. In microglial and mixed glial contexts, protective outcomes appear to depend on recipient cells with sufficient degradative and metabolic capacity to process incoming aggregates. Whether this capacity is preserved during chronic neuroinflammation, advanced age, or sustained proteotoxic stress remains unclear. If aggregate protein burden exceeds local clearance capacity, TNT-mediated transfer may become less effective as a compensatory mechanism and could instead contribute to pathological redistribution across compromised glial and microglia–neuron networks. Defining the conditions that distinguish TNT-mediated rescue from maladaptive cargo redistribution in the aging and diseased brain remains an important challenge for the field.

## Dendritic nanotubes in neuron-to-neuron connectivity

DNTs represent a newly uncovered, short-range layer of neuronal connectivity in the mammalian brain. Whereas TNTs have been described across diverse cell types and biological contexts, DNTs appear to represent a structurally distinct form of connectivity between dendrites in the mammalian cortex (Chang et al., 2025). Using super-resolution radial fluctuation microscopy, volumetric deconvolution, and machine learning, investigators identified ultra-thin, F-actin-rich membranous bridges linking dendrites of neighboring neurons in mouse and human cortical tissue (Figure 1C). DNTs were relatively short, spanning only a few microns, and appeared more reminiscent of dendritic filopodia than the longer TNTs (≥10 microns) described *in vitro*. However, this difference could reflect structural constraints imposed by the dense CNS environment. Electron microscopy confirmed that DNTs form short closed-ended membranous contacts between adjacent dendrites rather than long open-ended conduits. Their capacity to mediate Ca^2+^ signal propagation and amyloid-β transfer suggests dual roles in physiological signaling and early pathological protein redistribution in the cortex.

Cross-sectional analysis of the medial prefrontal cortex in APP/PS1 mice, an Alzheimer’s disease model of amyloid pathology, suggests that DNT networks are dynamically altered during early amyloid pathology (Chang et al., 2025). DNT density increased at 3 months of age, before overt plaque deposition, but declined by 6 months as pathology progressed. This biphasic trajectory raises the possibility that DNTs are initially upregulated in response to early amyloid-associated stress but later diminish as disease burden escalates. Complementary computational modeling further suggests that DNT-mediated amyloid-β transfer may contribute to early amyloid accumulation.

Despite these advances, fundamental mechanistic questions remain unresolved. Progress is limited in part by the lack of specific molecular markers for DNTs and absence of information regarding the biophysical basis of cargo transfer across DNT junctions. One plausible mechanism is that small signaling molecules are transmitted through junctional coupling at DNT contact sites. In non-neuronal cell lines, TNTs mediated electrical coupling via connexin-containing gap junctions at the TNT-target cell interface (Wang et al., 2010). Similar work suggests that a second messenger, inositol 1,4,5-trisphosphate (IP3), rather than Ca^2+^ itself, diffuses across these junctions to trigger Ca^2+^ release from ER stores in the receiving cell (Lock et al., 2016). Whether an analogous or novel mechanism operates at DNT junctions remains an intriguing question for the field.

Intercellular transfer of macromolecular cargo such as amyloid-β is more difficult to reconcile with a closed-ended architecture. Although Chang et al. (2025) demonstrated that amyloid-β transfer requires intact, F-actin-based DNTs and argued against passive extracellular vesicle uptake, the exact mechanics of this transfer are unresolved. Possibilities such as transient membrane fusion, localized endocytosis at contact sites, or surfing of oligomers along the DNT surface remain to be explored. Functional and mechanistic evidence for DNTs was derived largely from young neuronal cultures and early-stage amyloid models. Clarifying how DNTs mediate Ca^2+^ signaling and amyloid-β transfer, as well as determining whether these functions persist in the aged or diseased human brain, are important next steps.

## From *in vitro* discovery to *in vivo* context

Much of the foundational understanding of nanotubular communication originated from *in vitro* models. Early studies using immortalized cell lines and primary cultures established that TNTs could mediate intercellular spread of neurotoxic proteins, including misfolded, self-propagating prion protein aggregates (Gousset et al., 2009), α-synuclein aggregates between cultured astrocytes (Rostami et al., 2017), and pathological tau assemblies between remote cells (Abounit et al., 2016). While these systems demonstrate that F-actin-based conduits transport pathological cargo, extrapolating to the intact brain requires caution. Cells grown on rigid, two-dimensional substrates experience markedly different structural and mechanical constraints than cells embedded within living tissue, and these simplified environments lack the dense extracellular matrix (ECM), three-dimensional architecture of the neuropil, and full complexity of multicellular interactions in the CNS (Kotarba et al., 2024). Thus, the rapid and transient formation of TNTs observed in isolated cultures may not fully reflect their behavior *in vivo*.

Recent work has begun to narrow this translational gap by integrating live-cell approaches with *ex vivo*, *in vivo*, and ultrastructural techniques. After engraftment of α-synuclein-loaded primary cortical neurons into mouse cortex, *in vivo* two-photon imaging showed α-synuclein transfer from neuronal somata to microglia, although TNT-mediated transfer could not be definitively resolved. Serial-section electron microscopy and tissue staining provided complementary evidence for TNT-like neuron–microglia contacts in brain tissue (Scheiblich et al., 2024). Super-resolution imaging and tissue-clearing approaches have uncovered neuron-to-neuron DNTs in the murine brain, with additional ultrastructural images from human cortical datasets (Chang et al., 2025). An investigation in the peripheral nervous system extends nanotubular glia–neuron interactions beyond the CNS. Strikingly, satellite glial cells transfer mitochondria to dorsal root ganglion sensory neurons *in vitro*, *ex vivo*, and *in vivo* through myosin X-dependent TNTs (Xu J. et al., 2026). Scanning and transmission electron microscopy also revealed TNT-like structures between these cells in mouse and human dorsal root ganglia. This mitochondrial transfer pathway was impaired in diabetic and chemotherapy-induced peripheral neuropathy models, indicating that glia-to-neuron mitochondrial rescue through TNTs can be disrupted in disease states. These studies collectively extend the field beyond simplified culture-based observations and show that nanotubular connections can be detected and studied within complex neural tissue.

Most *in vivo* and tissue-based studies to date rely on young adult tissue or early-stage transgenic disease models, leaving it unclear whether comparable nanotubular networks are formed, maintained, and remain functionally effective in aged neural tissue. The aging microenvironment introduces substantial mechanical and biochemical changes, including ECM stiffening, chronic low-grade neuroinflammation, and accumulation of dysfunctional glia (Gao et al., 2023; Lai and Wong, 2020), which may alter the formation, stability, and/or function of fragile nanotubular structures. How these age-related shifts influence nanotubular integrity and the cell-type-dependent balance between protective rescue and pathological cargo redistribution remains an important unresolved question.

## Aging as a potential modulator of nanotubular dysfunction

While ongoing work has begun to uncover nanotubular networks in early disease contexts, how these systems change across the natural lifespan remains to be determined. Current models often emphasize pathological aggregates such as amyloid-β as major disruptors of nanotubular connectivity. Here, we consider chronological aging as an underexplored modifier of nanotubular function, one that may alter network stability and cell-type-specific vulnerability before late-stage pathology emerges.

Age-associated changes in actin dynamics and cytoskeletal integrity are well described (Lopez-Otin et al., 2013; Lai and Wong, 2020). If the structural scaffolding required for TNT formation and maintenance becomes less stable with age, the brain may become increasingly less effective at sustaining protective intercellular exchange. Genetic risk variants may further compromise TNT integrity or function. Previously discussed TREM2 and LRRK2 variants can compromise TNT-mediated cargo handling and microglial support of aggregate-burdened cells, raising the possibility that aging and genetic risk converge on shared vulnerabilities in TNT rescue mechanisms.

The aging microenvironment may also reshape TNT function through changes in the surrounding milieu. Chronic low-grade neuroinflammation, or inflammaging (Franceschi et al., 2018), may alter TNT formation, stability, and/or cargo handling. Prior work revealed that pro-inflammatory signaling primes dendritic cells to form TNT networks during antigen presentation to T helper cells, supporting antigen exchange between dendritic cells while also permitting surfing of extracellular pathogens, e.g., HIV-1 (Zaccard et al., 2015; Zaccard et al., 2016). Although comparable inflammation-driven regulation has not been established in the aging brain, inflammaging could alter TNT-mediated aggregate clearance, mitochondrial rescue, or pathological cargo redistribution between connected cells.

Together, age-related cytoskeletal changes, genetic risk factors, and inflammaging may alter nanotubular function before overt structural collapse, affecting network stability, directionality, or cargo-handling capacity. Mapping nanotubular connectivity across brain regions, cell types, and lifespans will be important for distinguishing protective rescue, impaired transfer, and maladaptive cargo redistribution in aging and neurodegenerative disease. This perspective may also broaden therapeutic strategies beyond targeting toxic aggregates alone, toward preserving beneficial intercellular support without enhancing pathological spread.

## Discussion

Recently identified nanotubular networks in the CNS suggest that intercellular communication depends on more than classical neural connections alone. Microglial and astrocytic TNTs, together with neuronal DNTs, point to a previously hidden layer of connectivity in which direct membranous bridges can support aggregate handling, metabolic rescue, and coordinated stress responses, while also permitting pathological protein transfer in a cell-type- and context-dependent manner. As illustrated by the rapid progression of the field over the past two decades (Figure 1D), nanotubular biology has moved from isolated *in vitro* observations to the identification of structurally and functionally distinct networks in intact neural tissue. A central challenge now is defining the conditions under which these networks support clearance or rescue versus pathological protein spread.

Aging remains an unresolved variable in this balance. Alongside pathological aggregates and genetic risk factors, chronological aging may alter nanotubular connectivity, stability, and/or function before overt collapse. The critical tipping point may therefore be functional rather than anatomical, reflecting changes in network directionality, cargo-handling capacity, or the ability of recipient cells to process incoming material. Rather than representing a uniform shift from protection toward dysfunction, this transition is likely governed by cell type, cargo burden, disease stage, and the metabolic or inflammatory state of connected cells. Characterizing these changes will require mapping TNT and DNT abundance, distribution, and function across brain regions and lifespans, particularly in aging and neurodegenerative disease models.

Progress may rely on expanding the field’s experimental toolkit. Identifying specific molecular markers for distinct nanotubular structures remains a major priority, as such markers would enable more precise tracking and mechanistic studies. Equally critical are imaging strategies capable of tracking fragile nanotubular networks across age and disease stages *in vivo* and in preserved tissue, alongside high-resolution ultrastructural analysis in diverse human postmortem cohorts. Clarifying how aging, chronic neuroinflammation, and genetic risk reshape nanotubular connectivity may reveal therapeutic strategies that preserve beneficial intercellular support while limiting pathological cargo redistribution.

## Data Availability

The original contributions presented in the study are included in the article/supplementary material. Further inquiries can be directed to the corresponding author.
